# The Electric Mallet

**Published:** 1910-05-15

**Authors:** B. J. Maercklein

**Affiliations:** Milwaukee, Wis.


### THE ELECTRIC MALLET.

BY B. J. MAERCKLEIN, MILWAUKEE, WIS.

The invention was originally the work of Bonwill but his first invention like those of so many others, was defective; it was so cumbersome and ineffective that it was practically worthless.

Dr. Marshall H. Webb took up the instrument as it existed, changed the size of it; made the magnets very much smaller; wound it with heavier wire and constructed it upon a more scientific basis and added very much more power to the battery to operate the instrument. This made it a very valuable instrument; it worked and gave a blow. The armature simply vibrated backward and forward.

The increase of battery power, difference in winding and the construction of the instrument in general, made it practically what it is to the present day. It has been changed somewhat, but the general appearance and construction is still the same. Now, the operation of the instrument is, in my opinion, the most perfect of any malleting instrument that has so far been devised, provided it is properly adjusted and properly handled. The ease with which it can be handled makes it practicable for you to use your hand in any position and in any direction that you please, while all other malleting instruments connected with an engine interfere, and render it impossible to give that delicacy of touch, which in my opinion, is one of the features necessary to make a perfect filling. The adjustment of the instrument is unfortunately not understood by many dentists. A very few are able to adjust it properly.

The instrument consists of a pair of horseshoe magnets to which on one side is attached a small rubber stem about four and one-half inches in length. This rubber stem is hollow and into it the mallet is carefully fitted and rests against a hard rubber plunger which is thrown back by a fine spring and propelled forward again by the armature. There is at the same time another small spring, which is interrupted by the force of the armature coming in contact with, or almost in contact with the magnets. This breaks the circuit, thereby making the instrument work automatically, so that when you once have made the circuit, the making and breaking of the circuit causes the armature to work in alternate directions and this in turn causes a very rapid and even blow.

Now, the manufacturers, not being operators, send out a large number of instruments that are very poorly adjusted. There is only a slight vibratory motion in this instrument and if the instrument is not properly adjusted, the working of the mallet is necessarily defective. Another point upon which they do not lay sufficient stress is that the interrupter rod should be so adjusted that it will only make a fair interruption, allowing the armature to get as closely to the ends of the magnets as possible, without actually coming in contact. This will give the most powerful blow, because the closer the armature gets to the magnets, the more powerful the attraction will be, while at a distance the attraction is a great deal less. The spring holding the plugger back against the armature should be so adjusted that whenever an impact is given by the armature to the plugger point, it will move forward and then recoil backward say, probably a thirty-second of an inch. When so adjusted the instrument will respond to the touch of the operator every time he moves his finger forward to make the connection. It gives the operator the most perfect control and ease that I have ever seen. It beats the engine; it beats the hand mallet, on account of its uniformity of blow. The blow can be regulated so that it is very slight or very heavy as the operator may desire by simply handling one screw and giving the armature more pull to come in on the blow. The operator, in filling with the electric mallet and perhaps with all other mechanical mallets, should not rest the point of the instrument solidly against the filling. If he does, then the blow will be a heavy thump and it will jar the tooth in its socket. If, however, the instrument is held back about a thirty-second of an inch, it will give an elastic blow to the tooth, strike it and then bound back. Now, every tooth has a certain amount of elastic resilience, and the tooth will not be injured by the lagest filling. If you do that with any other instrument, you will thump the tooth in the socket. This is one of the essential features of the mallet.

Now, in addition to the condition of the plugger points there is only one thing in which there is a large divergence of opinion in regard to all kinds of malleting points. On all malleting plugger points the serrations ought to be very fine and the serrations ought to be at right angles.

You may say that the force of all blows travels in a direct line, and cannot be changed from that line until some other force changes it or re-directs it. You must, therefore, prepare your cavities in such a manner that you can reach the bulk of the cavity with your mallet if you are going to use mallet force. The hand may be made to follow the curve of the cavity and you can follow from the posterior surfaces forward, but you must bend the instrument and put it in the cavity and follow that curve. The force will be in the direction taken by the instrument.

With these few points in mind and an instrument properly adjusted, you can do work with it as with no other instrument. It takes the place of the finest office assistant that you can possibly obtain for malleting. The blow being uniform and under the control of the operator, you can regulate it with ease.

I regret that I have not been able, under the circumstances, to write out a more lengthy argument on this subject and read it to you, as the instrument is, in my estimation, deserving of it. I have tried nearly all other instruments. I find that in my hands it has no equal. Whatever has been said that it is a very slow working instrument, it has been repeatedly demonstrated that you can put in more gold into a cavity in a limited amount of time with this instrument, than with any other instrument. Dr. Marshall H. Webb years ago made a number of tests; in one of these he put in 43 grains of gold in 40 minutes time. Another man filled in 24 grains of gold in 23 minutes time. It remains for the profession to become more expert in handling the instrument and to learn how to use it. When you have once acquired the exact knowledge you would about as soon quit dentistry as be without the instrument.

